# Lockdown stringency and employment formality: evidence from the COVID-19 pandemic in South Africa

**DOI:** 10.1186/s12651-022-00329-0

**Published:** 2023-01-11

**Authors:** Timothy Köhler, Haroon Bhorat, Robert Hill, Benjamin Stanwix

**Affiliations:** Development Policy Research Unit, School of Economics, University of Cape Town, Cape Town, South Africa

**Keywords:** South Africa, Lockdown, Lockdown stringency, COVID-19, Labour market, Formality, D04, J08, J20, J48, J88

## Abstract

In response to COVID-19 most governments used some form of lockdown policy to manage the pandemic. This required making iterative policy decisions in a rapidly changing epidemiological environment resulting in varying levels of lockdown stringency over time. While studies estimating the labour market effects of lockdown policies exist in both developed and developing countries, there is limited evidence on the impact of variation in lockdown stringency, particularly in developing countries. Such variation may have large heterogenous effects both on aggregate and between worker groups. In this paper, we estimate the causal effect of lockdown stringency on employment probabilities, adopting a quasi-experimental design on unique labour force panel data from South Africa. South Africa is a useful case study given its upper-middle-income status and relatively small informal sector, thus serving as an example to a variety of developing and developed country economies. We find that the negative employment effects of the country’s lockdown policy were driven by effects on the informal sector. Furthermore, we observe important effect heterogeneity by employment formality as the stringency of the country’s lockdown regulations changed over time. We find that more stringent lockdown levels negatively affected informal, but not formal sector employment, while less stringent levels negatively affected formal, but not informal sector employment. From a policy perspective, evidence of such heterogeneity can inform decisions around the optimal targeting of support as the pandemic progresses and lockdown policies are reconsidered.

## Introduction

Like many governments around the world, in response to the COVID-19 pandemic the South African government implemented a national lockdown to prepare necessary health infrastructure as well as delay and minimise the spread of the virus. This initial lockdown, which began on 27 March 2020 and lasted for 5 weeks, was relatively stringent by international standards (Bhorat et al. 2020a, b; Gustafsson 2020). The regulations prevented any non-essential activities outside the home, imposed restrictions on all public gatherings, led to the closure of all schools, the introduction of a curfew, a prohibition on the sale of tobacco products and liquor, and strict domestic and international travel controls. Research using anonymized mobile phone data shows a substantial reduction in population mobility in response to these regulations (Carlitz and Makhura 2021). In the labour market, only workers in occupations deemed essential for economic function and pandemic response were permitted to continue working at their usual place of work during this period. A lockdown of this type was always expected to have significant economic costs, and official estimates reveal a contraction of 2.2 million jobs (14%) in the second quarter of 2020 relative to the first—essentially erasing the last decade of job growth in the economy.

Crucially, the job axe of this initial lockdown did not fall evenly. In line with the global literature, there was an unequal distribution of job loss by employment formality in South Africa (Benhura and Magejo 2020; Rogan and Skinner 2020; Köhler et al. 2021). Informal sector workers accounted for approximately 50% of all net jobs lost in the short-term, despite these workers representing just 25% of total pre-pandemic employment (Köhler et al. 2021). Such a disproportionate incidence of job loss has been observed across Sub-Saharan Africa (Balde et al. 2020; Schotte et al. 2021), and it has been argued that the characteristics of informal sector jobs—including a higher propensity of being in contact-intensive industries, lower propensities of being able to work remotely, and fewer legal protections such as paid leave and unemployment insurance—likely explain these adverse outcomes (Fox and Signe 2020; ILO 2020; Ngameni 2020). Whatever the mechanism, informal sector employment has served as a key predictor of job loss during South Africa’s initial lockdown period.

Lockdown policy in South Africa and around the world was, however, not time-invariant. Indeed, governments continue to make policy decisions in a context of significant uncertainty and a swiftly changing epidemiological situation, resulting in varying levels of lockdown stringency. In South Africa, following the initial ‘hard’ lockdown described above, the government adopted a five-level strategy, with lockdown stringency varying according to the severity of contagion. It is plausible that such variation may have heterogenous effects both on aggregate and by employment formality. Although studies providing evidence on the labour market effects of the pandemic and lockdown policies exist in both developed countries^1^ and few developing countries,^2^ there is a lack of causal evidence on how variation in lockdown stringency affects labour market outcomes, both on aggregate and by employment formality. From a policy perspective, evidence of such heterogeneity not only provides a useful retrospective analysis, but also has the potential to inform future decisions about lockdown regulations and the optimal targeting of government support.

In this paper, we estimate the causal effect of lockdown stringency on employment probabilities and examine effect heterogeneity by employment formality. We do so in the context of South Africa, which serves as a useful case study for two key reasons: (i) the timing of changes in the country’s lockdown levels coincide with labour forces survey periods, which allows us to accurately isolate the effect of varying levels of lockdown stringency, and (ii) as an upper-middle-income country with a relatively small informal sector employment share, our findings may be broadly useful to both developing countries (given South Africa’s level of economic development) as well as to more developed countries (given South Africa’s low informal sector employment share^3^). Our analysis uses representative, individual-level, panel labour force data, and adopts a quasi-experimental econometric design to exploit temporal variation in employment probabilities of adults who were and were not permitted to work. We consider several levels of lockdown stringency over time and explore effect heterogeneity by employment formality. Specifically, we cross-reference South Africa’s lockdown regulations to over 150 industry codes in the data, and make use of the coincidental timing of the onset of the lockdown and data collection periods to estimate a Difference-in-Differences (DiD) model. We further exploit the panel nature of the data to control for observable and unobservable time-invariant heterogeneity through individual fixed effects.

We find that while South Africa’s lockdown policy significantly reduced employment probabilities for every level of lockdown stringency, these effects were driven by negative employment effects in the informal sector. Moreover, these effects were heterogeneous by lockdown stringency—where more stringent lockdown levels negatively affected informal sector employment but not formal sector employment, while the least stringent lockdown level negatively affected formal sector employment but not informal sector employment. Overall, we estimate that South Africa’s lockdown policy decreased the probability of employment for those not permitted to work by 3 percentage points, or 36.15% relative to the control group; however, this effect was driven by a negative effect in the informal sector (2.6 percentage points). We find no evidence of such an effect on formal sector employment. Considering lockdown stringency, we estimate significant negative effects of 3.4–3.5 percentage points on informal sector employment probabilities for the most stringent lockdown levels; however, we find no evidence of any such effects for the least stringent level. In contrast, we estimate a significant and negative 6.1 percentage point effect on the probability of formal sector employment for this least stringent lockdown level but find no evidence for such effects for more stringent lockdown levels. These results hold when subject to robustness tests relating to placebo outcomes, the possibly confounding effect of varying task content across occupations with respect to physical interaction, which we control for by merging in task content data from the Occupational Information Network (O*NET) and generating a physical interaction index, as well as varying treatment group assumptions.

We hypothesize two potential reasons for the heterogeneous relationship between lockdown stringency and formality of employment. First, we posit that this finding may be related to between-sector variation in employment elasticities with respect to ‘abrupt’ versus ‘accumulated’ lockdown effects. Second, we discuss the plausibility that such heterogeneity may be explained by a combination of differential targeting and timing of two of the government’s core economic support policies during the beginning of the pandemic: a wage subsidy which temporarily targeted primarily formal sector workers but later included informal sector workers, and a new unconditional cash transfer which, in addition to supporting the unemployed, provided support to informal sector workers but which was only largely rolled out during the least stringent lockdown level assessed here. The differential timing and targeting of these policies, coupled with our findings, suggest that they may have mitigated the negative employment effects of the country’s lockdown policy. However, a detailed mediation analysis lies beyond the scope of this paper and as such we are unable to make conclusive statements in this regard, but an empirical analysis of these potential mechanisms serves as an important area for future research. Overall, our analysis highlights the differential effects of lockdown policies by level of stringency and employment formality. As the pandemic progresses and governments continue to consider lockdown restrictions as a policy response, policymakers ought to consider the existence of such heterogeneous effects by lockdown stringency and employment formality in their efforts to target support appropriately.

The remainder of this paper is structured as follows. Section 2 first provides an overview of South Africa’s national lockdown policy and how policy stringency varied over the course of the pandemic, as well as an overview of the economic policy package the government introduced to provide support to vulnerable firms, workers, and households. Thereafter, we provide a description of the dataset and the balanced panel sample. In Sect. 3 we describe our identification strategy and model specification. We present our results in Sect. 4 and thereafter the robustness tests in Sect. 5. Finally, in Sect. 6 we conclude.

## Context and data

### COVID-19 lockdown regulations in South Africa

**Fig. 1:**
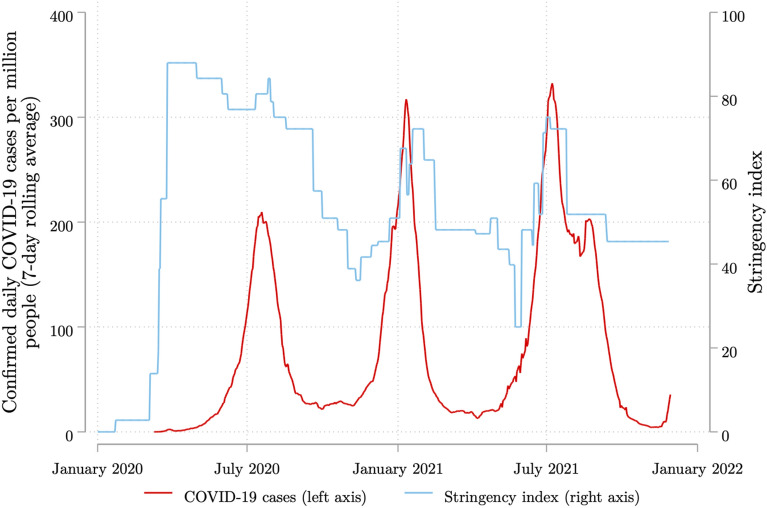
Trajectories of COVID-19 cases and lockdown stringency in South Africa, 2020 and 2021. *Author’s own arrangement. * *Source*: Our World in Data (Ritchie et al. 2020); Oxford Covid-19 Government Response Tracker (OxCGRT) dataset (Hale et al. 2020). *Notes*: This figure presents the 7-day rolling average of confirmed daily COVID-19 cases and the government policy stringency index values over time. The stringency index measures the strictness of policies that restrict people’s behaviour, calculated using data on nine indicators of government containment and closure policy and public information campaigns. The index ranges from 0 to 100 with higher scores indicative of stricter policy responses

Following the report of the first confirmed COVID-19 case in South Africa on 5 March 2020, the government implemented a national lockdown that began on 27 March 2020. This initial lockdown lasted until the end of April 2020 and was relatively stringent by international standards (Gustaffson 2020), making no allowances for any non-essential activity outside the home. All schools were closed, a curfew was enforced, and only workers in occupations deemed essential for economic function and pandemic response were permitted to work—for example: healthcare workers, emergency personnel, security services, utilities, telecommunications, and certain mining and agriculture sub-industries. Additionally, the sale of alcohol and tobacco products was forbidden, with the latter regulation making the country one of only three in the world to do so (Van Walbeek et al. 2021). Estimates using pre-pandemic data suggest that during this period just 40% of the employed were officially permitted to work (Francis et al. 2020). In Fig. 1, we plot trends in the weekly rolling average of confirmed daily COVID-19 cases and a government policy stringency index over 2020 and 2021. The stringency index is sourced from the Oxford COVID-19 Government Response Tracker (OxCGRT) dataset and is a composite measure of the strictness of policies that restrict people’s behaviour, calculated using data on nine indicators of government containment and closure policy (including school and workplace closures, restrictions of public gatherings, stay-at-home requirements, and domestic and international travel controls) (Hale et al. 2020).^4^ A higher index score is indicative of a stricter policy response. It is clear that government policy stringency was highest during this initial lockdown period, given the index score of 87.96 during April 2020—the highest value for South Africa since the onset of the pandemic.

Following this initial lockdown the government adopted a five-level risk-adjusted strategy which implemented lockdown regulations, still at the national level, according to the severity of contagion, with the first ‘hard’ lockdown period regarded as level 5. From May 2020 the country moved to level 4, which permitted all agricultural activities and a limited group of manufacturing, construction, and mining activities, but at reduced capacities. Restaurant services were permitted, but only in the form of delivery services for off-site consumption during limited times and subject to the curfew. The sale of alcohol and tobacco products remained banned. From June 2020, level 3 regulations were in place and permitted almost all sectors to operate except for tourism, hospitality, and several entertainment industries whose activities remained highly restricted and, in some cases, prohibited. Although the tobacco sales ban persisted until August 2020, the alcohol sales ban was lifted; however, trading hours were limited, and sales were only permitted for off-site consumption. In the education sector, a phased re-opening of schools was adopted with only learners in specific grades being allowed to attend school, while attendance for all grades was permitted from 31 August 2020.^5^ The gradual easing of the regulations from level 5 in April 2020 to level 3 from June 2020 can be observed in Fig. 1, with the stringency index score reducing to 84.26 during level 4 and 76.85 during level 3. Thereafter, lockdown stringency varied as the pandemic progressed but closely followed the trajectory of confirmed cases and continued to be implemented at the national level. At the time of writing, the government’s five-level risk-adjusted strategy remained in place.

### The South African government’s economic support policy package

In response to the pandemic and national lockdown regulations, the South African government introduced a broad economic policy package in March and April 2020 to provide largely cash-based relief to two broad groups: firms and households. This package, initially amounting to 10% of Gross Domestic Product (GDP), primarily consisted of tax relief measures and a combination of existing and new social protection and labour market programmes, many of which were extended and revised as the pandemic progressed and lockdown regulations varied (Bhorat et al. 2020a, b; Gronbach et al. 2022). Collectively, these policies targeted relief to firms and workers in both the formal and informal sectors, as well as the unemployed and individuals residing in poor households.

Regarding support to the latter group, the primary programmes leveraged off the country’s large, non-contributory social assistance infrastructure and included an expansion on both the intensive and extensive margins: a temporary increase in the value of all existing unconditional cash transfers, which benefitted over 18 million recipients as well as their co-residents in poor households; and the introduction of a new ‘COVID-19 Social Relief of Distress’ (SRD) grant of ZAR350 per person per month (US$49 in Purchasing Power Parity (PPP) terms) which provided an income source to unemployed adults who received no other form of government support. Although the grant explicitly targeted the unemployed population, Köhler and Bhorat (2021) show that by approximately 6 months into the grant’s roll-out up to 40% of recipients of the grant were informally employed. Such inclusion error was not however unexpected given the government’s capacity to distinguish the unemployed from the informally employed and verify eligibility, and in fact the grant was initially conceptualised to target the informally employed (Bassier et al. 2021). As the pandemic progressed the grant experienced several extensions and, by the end of 2021, had brought over 10 million previously unreached adults into the system (SASSA 2022). Several observational studies have highlighted the grant’s large coverage, progressive distribution, and (simulated) positive effects on welfare (Köhler and Bhorat 2020, 2021; Bassier et al. 2021, 2022; Bhorat et al. 2021; Barnes et al. 2021; van der Berg et al. 2022; Turok and Visagie 2022). At the time of writing, the grant still remains in place but was scheduled to be terminated in March 2023.

The dominant policy which targeted support to firms and workers in the formal sector, which accounts for the majority of the employed in South Africa, was a wage subsidy scheme. Formally referred to as the COVID-19 Temporary Employer-Employee Relief Scheme (TERS), the policy provided relief to workers who suffered income loss because of a full or partial closure of their employer’s operations, and had the primary aim of mitigating job loss. Considering South Africa’s extreme levels of unemployment, Köhler and Hill (2022) argue that such a job retention policy has served as the country’s most important labour market intervention in response to the pandemic. Benefits ranged from 38 to 60% of a worker’s wage subject to lower and upper limits, or between ZAR3 500 (US$ 502 PPP, the equivalent of the national minimum wage) and ZAR6 730 (US$966 PPP) per month. To implement the policy, the government used existing structures, databases, and legislation and, as such, was able to provide support to workers and firms both timeously and effectively without the need for a special registration drive (Gronbach et al. 2022). The TERS also experienced various extensions and revisions as the pandemic progressed, and by 2 years after its inception, over 5.7 million workers had benefitted (Nxesi 2022). Existing studies have provided favourable evidence of the positive effects of the policy on job retention during the beginning of the pandemic, both descriptively (Köhler and Hill 2022) and causally for formal, private sector workers (Köhler et al. 2022). At the time of writing, the policy was drawing to a close.

### Data

#### The quarterly labour force survey

The analysis in this paper uses individual-level survey data from Statistics South Africa’s (StatsSA) Quarterly Labour Force Surveys (QLFS)—the country’s official source of labour market statistics—for the first two quarters of 2020. The QLFS is a cross-sectional (with a rotating panel element), nationally representative household survey, conducted every quarter since 2008, that contains detailed information on a wide array of demographic and socioeconomic characteristics and labour market activities for individuals aged 15 years and older. The survey follows a stratified two-stage sampling design, with probability proportional to size sampling of primary sampling units (PSU) in the first stage and sampling of dwelling units with systematic sampling in the second stage (Statistics South Africa 2008). As such, the sampling unit is the dwelling and the unit of observation is the household. The sampling weights for the data account for original selection probabilities and non-response and are benchmarked to known population estimates of the entire civilian population of South Africa. We use these weights throughout the analysis unless specified otherwise and restrict the sample to the working-age population (aged 15–64 years).

#### Pandemic-induced changes to the QLFS

There are several important differences in the 2020 QLFS data that are worth noting in some detail here. Prior to the pandemic, the QLFS sample consisted of nearly 70,000 individuals living in approximately 30,000 dwelling units, with data being collected via face-to-face interviews. However, towards the end of March 2020, StatsSA suspended face-to-face data collection, resulting in 621 sampled dwelling units (2% of the sample) were not interviewed in the quarter 1 dataset. To adjust for this, StatsSA used the rotational panel component of the survey to make imputations where possible using data from the previous quarter. To continue providing labour market statistics for the remainder of the year, StatsSA changed its data collection mode to computer-assisted telephone interviewing (CATI). To facilitate this, and unlike in previous quarters, the sample that was surveyed in 2020Q1 and for which StatsSA had contact numbers was surveyed again in 2020Q2. The result was that the 2020Q2 data included the majority (71%) of the 2020Q1 sample, as not all dwelling units had contact numbers.^6^ Unlike the data prior to the pandemic, this sampling decision hence resulted in the survey changing from a cross-sectional survey with a rotational panel element to an (unbalanced) panel survey—a novel scenario in the survey’s history. We exploit this aspect of the data in our identification strategy, detailed below.

The concern here is that this sampling decision may produce 2020Q2 estimates that suffer from selection bias given characteristic differences between ‘telephone’ and ‘non-telephone’ households. To address this source of bias, StatsSA adjusted the calibrated survey weights using several bias-adjustment factors (Statistics South Africa 2020a). At the time of writing, an explicit external review of this procedure has yet to be conducted and would require more information than is available in the public QLFS documentation. Table 8 in the “Appendix” presents an overview of the sample sizes and weighted population estimates of several labour market groups by quarter. We additionally include the relevant estimates for the same period 1 year prior (2019) for comparison. These estimates suggest the bias-adjusted weights appear to be appropriately computed. From an unweighted sample of over 66,000 individuals, the South African population estimate in 2020Q1 of 57.8 million is not economically or statistically significantly different from the 2020Q2 estimate, despite the latter sample consisting of nearly 20,000 fewer observations. In contrast, the weighted estimates of specific labour market groups are statistically significantly different in size between quarters, which is expected given the onset of the pandemic. These differences between quarters in 2020 are similar to the year-on-year differences within quarter two, and considering the first quarter, the between-year sample sizes are similar, and the weighted estimates are not statistically significantly different from one another, apart from the broad unemployed sample size and population estimate which have grown by 6% and 8% year-on-year, respectively. The smaller samples from 2020Q2 result in expectedly less precise estimates, as reflected by the larger standard errors. Despite the above similarities, it should be noted that StatsSA’s bias-adjustment procedure relied on observable characteristics such as age, gender, and race; however, respondents may still be unobservably different from non-respondents and hence possibly from the broader population. The possibility of this outcome was confirmed by StatsSA through a telephone interview (Statistics South Africa 2021, personal communication, 2 September). At the time of writing, an explicit external review of the construction of these weights has yet to be conducted and would require more information than is available in the public QLFS documentation.

#### Balanced panel sample representivity

Treatment assignment in our identification strategy, detailed below, is a function of an individual’s pre-pandemic (2020Q1) industry, and therefore relies on observing individuals in both time periods. As such, we restrict our analysis to the balanced panel sample of individuals observed in both periods. Given that the QLFS is usually used as a cross-sectional survey described above, we make use of household and person identifiers in the data to ensure we observe the same individual over time. However, even after doing so, we observe several instances where a given individual has the same household and person identifier between quarters but varies in other characteristics which plausibly should not exhibit such variation (for example, age changing from 41 years in 2020Q1 to 58 years in 2020Q2). To address this, although the anonymity of the data prohibits us from accessing other identifying variables such as names and surnames, in addition to household and person identifiers we make use of data on age (years), gender, and self-reported racial population group to identify the same individual over the period. While the latter two characteristics should be time-invariant, we allow for a 1-year difference in age between 2020Q1 and 2020Q2 in either direction to account for ageing or measurement error. This procedure results in a balanced panel sample of 24,475 unique working-aged (as of 2020Q1) individuals, each observed twice in the data (equivalent to 48,950 observations in total).

To determine whether this sample remains representative of the larger population, we estimate means for several observable covariates as well as our three outcome variables in the baseline period (2020Q1) for the cross-sectional and balanced panel samples and conduct *t*-tests to determine whether any observed differences in means are statistically and economically significant. We additionally include the relevant estimates for the same vector of covariates in the same period 1 year prior (2019Q1) for comparison. We present these estimates in Table 1. Considering the statistically significant differences, individuals in our panel sample appear more likely to be older, female, African/Black, and have a post-secondary education, while being less likely to live in an urban area and have a highest education level of primary or less. Despite the statistical significance of these differences, they can be said to be economically insignificant given that their magnitudes are all relatively close to zero. This finding holds when considering differences between the balanced panel sample and either the cross-sectional estimates from 2020Q1 or 2019Q1. We are therefore confident that our sample remains relatively representative of the broader South African working-age population.

**Table 1:** Covariate balance table at baseline, by sample. *Author’s own calculations. Source*: QLFS 2019Q1, 2020Q1, and 2020Q2 (Statistics South Africa 2019a, b, 2020b, c)

Sample	(1)	(2)	(3)	(1)–(3)	(2)–(3)
	Cross-sectional		Balanced panel	Difference	
Period	2019Q1	2020Q1	2020Q1		
Observations	42,024	41,827	24,475		
*Demographics*					
Age (years)	34.890	35.040	35.328	− 0.437***	− 0.287***
	(0.070)	(0.070)	(0.086)	(0.110)	(0.054)
Female	0.505	0.505	0.515	− 0.010***	− 0.011***
	(0.002)	(0.002)	(0.003)	(0.004)	(0.002)
African/Black	0.806	0.808	0.829	− 0.023***	− 0.020***
	(0.005)	(0.005)	(0.006)	(0.006)	(0.004)
Urban	0.677	0.680	0.658	0.019**	0.022***
	(0.005)	(0.005)	(0.007)	(0.008)	(0.005)
Married	0.355	0.350	0.353	0.001	− 0.004
	(0.004)	(0.004)	(0.004)	(0.005)	(0.003)
Primary education or less	0.143	0.134	0.127	0.016***	0.007***
	(0.002)	(0.002)	(0.003)	(0.003)	(0.002)
Incomplete secondary education	0.435	0.433	0.433	0.002	0.000
	(0.003)	(0.003)	(0.004)	(0.005)	(0.002)
Complete secondary education	0.297	0.306	0.308	− 0.011**	− 0.001
	(0.003)	(0.003)	(0.004)	(0.005)	(0.002)
Tertiary education	0.125	0.127	0.132	− 0.007*	− 0.005***
	(0.003)	(0.003)	(0.003)	(0.004)	(0.002)
*Occupation and sector*					
Legislators and managers	0.076	0.075	0.076	0.000	− 0.002
	(0.002)	(0.002)	(0.003)	(0.004)	(0.002)
Professionals	0.047	0.050	0.053	− 0.005*	− 0.003**
	(0.002)	(0.002)	(0.003)	(0.003)	(0.001)
Technical and associate professionals	0.080	0.077	0.080	0.000	− 0.004**
	(0.002)	(0.002)	(0.003)	(0.003)	(0.002)
Clerks	0.104	0.103	0.105	− 0.001	− 0.002
	(0.003)	(0.002)	(0.003)	(0.004)	(0.002)
Service and shop workers	0.163	0.167	0.171	− 0.008*	− 0.004*
	(0.003)	(0.003)	(0.004)	(0.005)	(0.002)
Skilled agricultural and fishery workers	0.003	0.004	0.004	− 0.001**	0.000
	(0.000)	(0.000)	(0.001)	(0.001)	(0.000)
Craft and related trades workers	0.127	0.127	0.125	0.002	0.002
	(0.003)	(0.003)	(0.003)	(0.004)	(0.002)
Plant and machine operators	0.082	0.081	0.085	− 0.003	− 0.004***
	(0.002)	(0.002)	(0.003)	(0.003)	(0.002)
Elementary workers	0.249	0.249	0.235	0.014***	0.015***
	(0.004)	(0.004)	(0.005)	(0.005)	(0.003)
Domestic workers	0.069	0.067	0.065	0.003	0.002
	(0.002)	(0.002)	(0.002)	(0.003)	(0.001)
Primary sector	0.080	0.079	0.067	0.012***	0.011***
	(0.003)	(0.003)	(0.003)	(0.004)	(0.002)
Secondary sector	0.212	0.210	0.213	0.000	− 0.002
	(0.003)	(0.004)	(0.004)	(0.005)	(0.002)
Tertiary sector	0.708	0.711	0.720	− 0.012**	− 0.009***
	(0.004)	(0.004)	(0.005)	(0.006)	(0.003)
*Province*					
Western Cape	0.121	0.121	0.108	0.013**	0.013***
	(0.004)	(0.004)	(0.005)	(0.005)	(0.004)
Eastern Cape	0.112	0.111	0.107	0.005	0.004
	(0.003)	(0.003)	(0.004)	(0.005)	(0.003)
Northern Cape	0.021	0.021	0.016	0.005***	0.005***
	(0.001)	(0.001)	(0.001)	(0.001)	(0.001)
Free State	0.050	0.049	0.048	0.001	0.001
	(0.002)	(0.001)	(0.002)	(0.003)	(0.002)
KwaZulu − Natal	0.185	0.185	0.212	− 0.027***	− 0.027***
	(0.004)	(0.004)	(0.006)	(0.007)	(0.004)
North West	0.068	0.068	0.062	0.005	0.005**
	(0.003)	(0.003)	(0.004)	(0.004)	(0.002)
Gauteng	0.269	0.270	0.253	0.016**	0.017***
	(0.005)	(0.004)	(0.006)	(0.007)	(0.004)
Mpumalanga	0.077	0.077	0.087	− 0.011***	− 0.011***
	(0.002)	(0.002)	(0.004)	(0.004)	(0.002)
Limpopo	0.098	0.098	0.106	− 0.008*	− 0.008***
	(0.003)	(0.003)	(0.004)	(0.005)	(0.003)
*Outcomes*					
Employed	0.426	0.421	0.422	0.004	− 0.001
	(0.003)	(0.003)	(0.004)	(0.005)	(0.002)
Employed in the formal sector	0.293	0.290	0.296	− 0.003	− 0.006**
	(0.003)	(0.003)	(0.004)	(0.005)	(0.002)
Employed in the informal sector	0.113	0.112	0.112	0.001	0.000
	(0.002)	(0.002)	(0.002)	(0.003)	(0.001)

This table presents estimates of mean values for observable covariates for the cross-sectional (either as of 2020Q1 or 2019Q1) and balanced panel sample (individuals observed in both 2020Q1 and 2020Q2; covariates values for this sample are as of 2020Q1) accompanied by difference estimates. Samples restricted to the working-age population (15–64 years). All estimates are weighted using sampling weights. Standard errors presented in parentheses and are clustered at the panel level. The magnitude and statistical significance of a given difference are estimated using *t*-tests

***p < 0.01, **p < 0.05, *p < 0.10

## Identification strategy

### Difference-in-differences

Our aim in this paper is to estimate the causal effect of a core component of South Africa’s lockdown—that which permitted certain individuals to work but other not—on employment probabilities. The ideal approach to establishing a causal effect entails randomised assignment of treatment where, in the context of this study, a given worker group was legally obligated to adhere to the regulations while another characteristically similar worker group was not. South Africa’s lockdown was of course not randomly assigned. It was implemented at the national level and as such legally obligated every worker to adhere to the regulations. However, being permitted to work was dependent on industry, as specified by legislation, which does provide a neat division of ‘treated’ and ‘untreated’ individuals over time. We cross-reference the lockdown regulations in the Government Gazettes with over 150 three-digit Standard Industrial Classification (SIC) codes available in the data to identify individuals who were and were not permitted to work. We then make use of the coincidental timing of the onset of the national lockdown and QLFS data collection periods to exploit across-group (treatment and control) and across-time (before and during the national lockdown) variation and estimate a Difference-in-Differences (DiD) model.^7^ Simply put, we estimate the causal effect of this core component of the lockdown by comparing employment probabilities between permitted-to-work and not-permitted-to-work worker groups from before to after the onset of the lockdown. It should be noted that this estimation strategy does not allow us to estimate the effects of South Africa’s lockdown policy in its entirety (that is, the cumulative effect of legislated permission-to-work policy, the curfew, school closures, and other restrictions on physical interaction and mobility), but rather the effect of one core component.

Our treatment group consists of all individuals who were legally not permitted to work while our control group consists of those who were. In Table 9 in the “Appendix” we present the categorised list of industries, at the 3-digit SIC level, by treatment status and lockdown level based on our cross-referencing procedure. Importantly, South Africa’s lockdown regulations were not time-invariant, as described above. To account for this, we make use of 2020Q2 interview month data, provided to us by StatsSA, which indicates whether an individual was surveyed in April, May, or June 2020. These periods fortunately coincide with changes in the lockdown levels, with level 5 in place from 1 to 30 April, level 4 from 1 to 31 May, and level 3 from 1 to 30 June in the 2020Q2 data. For example, individuals were included in the treatment group if they were not permitted to work under level 5 regulations and, in the post-treatment period, they were interviewed in April 2020 during level 5. Given that at the onset of the lockdown, this legislation affected individuals based on the industry they were already working in, treatment assignment for each observation in our analysis is a function of their pre-pandemic (2020Q1) industry. Therefore, our treatment variable is time-invariant within-individuals. In the pre-pandemic period, we have non-missing industry data for 13,143 of 24,475 observations, so as such we are only able to code treatment for 26,286 (13,143 multiplied by two) observations in total in the two-quarter period. In some instances, certain industries were permitted to operate but only at a limited employment capacity (these industries are indicated in Table 9). Our treatment assignment rule above would suggest coding these workers into the control group; however, at the firm-level they may not have been permitted to work given the capacity constraint. Although the data does not allow us to accurately identify these cases accurately, to address this we assign individuals in these ‘limited capacity’ industries to the control group if the legislated capacity was equal to or exceeded 50%. In our analysis, we use alternative thresholds to examine the sensitivity of our results to this assumption.

### Covariate balance and pre-treatment dynamics

The identifying assumption of our DiD approach implies that in the absence of the lockdown the trends of outcomes of those not permitted to work on average would have been similar to those permitted to work; in other words, the control group provides an appropriate counterfactual. Balanced mean levels of covariates or outcomes between the treatment and control group at baseline is not a requirement in a DiD strategy, however the validity of this design may be threatened if the difference in the mean levels of covariates (but not outcomes) varies significantly from before to after treatment. To examine this, in Table 2 we present estimates of means for all observable covariates used our models as well as our outcomes of interest both in the pre-lockdown and lockdown period by treatment status, as well as estimates of between-group differences both within and across periods. For the covariates, these latter between-group between-period estimates are equivalent to those obtained through placebo falsification tests where the DiD model is estimated separately on covariates which, in theory, should not be affected; for the outcomes, these estimates are equivalent to unconditional DiD estimates.

**Table 2:** Covariate balance table, by treatment status and period. *Author’s own calculations. Source*: QLFS 2020Q1 and 2020Q2 (Statistics South Africa 2020b, c)

Group	(1)	(2)	(3)	(4)	(5)	(6)	(7)
	2020Q1 (pre-lockdown)			2020Q2 (lockdown)			Diff-in-Diff: (6)–(3)
	Permitted to work (C)	Not permitted to work (T)	Diff: (2)–(1)	Permitted to work (C)	Not permitted to work (T)	Diff: (5)–(4)	
Observations	7620	5523		7620	5523		
*Covariates*							
Age (years)	39.567	39.432	− 0.135	39.652	39.645	− 0.007	0.128
	(10.647)	(11.016)	(0.209)	(10.679)	(10.938)	(0.217)	(0.095)
Female	0.476	0.470	− 0.007	0.464	0.460	− 0.005	0.002
	(0.499)	(0.499)	(0.010)	(0.499)	(0.498)	(0.010)	(0.003)
African/Black	0.779	0.797	0.018**	0.756	0.776	0.020**	0.001
	(0.415)	(0.402)	(0.008)	(0.429)	(0.417)	(0.009)	(0.003)
Urban	0.735	0.729	− 0.005	0.745	0.753	0.008	0.013
	(0.441)	(0.444)	(0.008)	(0.436)	(0.431)	(0.008)	(0.011)
Primary education or less	0.106	0.109	0.003	0.109	0.106	− 0.003	− 0.006
	(0.308)	(0.312)	(0.006)	(0.312)	(0.307)	(0.006)	(0.004)
Secondary education incomplete	0.343	0.370	0.027***	0.346	0.378	0.032***	0.005
	(0.475)	(0.483)	(0.009)	(0.476)	(0.485)	(0.010)	(0.006)
Secondary education complete	0.346	0.325	− 0.021**	0.348	0.325	− 0.023**	− 0.003
	(0.476)	(0.468)	(0.009)	(0.476)	(0.468)	(0.010)	(0.006)
Tertiary education	0.205	0.196	− 0.009	0.197	0.192	− 0.005	0.004
	(0.404)	(0.397)	(0.008)	(0.398)	(0.394)	(0.008)	(0.005)
Wage employment	0.855	0.865	0.009	0.775	0.766	− 0.010	− 0.019
	(0.352)	(0.342)	(0.007)	(0.417)	(0.424)	(0.008)	(0.014)
Industry or occupation job-mover	0.376	0.370	− 0.007	0.381	0.377	− 0.004	0.002
	(0.484)	(0.483)	(0.009)	(0.486)	(0.485)	(0.010)	(0.003)
Formality job mover	0.032	0.052	0.019***	0.032	0.051	0.019***	0.000
	(0.177)	(0.221)	(0.004)	(0.177)	(0.221)	(0.004)	(0.001)
*Outcomes*							
Employed	0.792	0.762	− 0.030***	0.670	0.612	− 0.058***	− 0.028***
	(0.406)	(0.426)	(0.008)	(0.470)	(0.487)	(0.010)	(0.008)
Employed in the formal sector	0.584	0.500	− 0.084***	0.501	0.427	− 0.075***	0.009
	(0.493)	(0.500)	(0.010)	(0.500)	(0.495)	(0.010)	(0.008)
Employed in the informal sector	0.165	0.260	0.095***	0.118	0.180	0.062***	− 0.033***
	(0.371)	(0.439)	(0.008)	(0.322)	(0.384)	(0.007)	(0.007)

This table presents estimates of mean values for observable covariates controlled for in our modelling to follow (excluding province for brevity) and outcomes by treatment group in the baseline and treatment periods accompanied by inter-group differences within and between periods for the balanced panel sample. Sample restricted to the working-age population (15–64 years). All estimates are weighted using sampling weights. Standard errors presented in parentheses and are clustered at the panel level. The magnitude and statistical significance of a given difference are estimated using *t*-tests

*T* treatment, *C* control, *Diff*. difference

***p < 0.01, **p < 0.05, *p < 0.10

We find that across most covariates, the mean levels for those permitted and not permitted to work within either period are not statistically significantly different from one another (see columns 3 and 6), and for the few that are, the magnitude and significance of the differences are stable from before to after the lockdown was introduced (see column 7). For instance, relative to those who would be permitted to work and in the pre-lockdown period, those that would not be permitted to work were approximately 2 percentage points more likely to be African/Black, 2 percentage points less likely to have a complete secondary education level, and just under 2 percentage points more likely to be a ‘formality job mover’ (defined as remaining employed over the period but transitioning from formal to informal sector employment or vice versa). The magnitudes and significance levels of all these differences in covariate values are similar during the lockdown period, and as indicated in column 7, the change in magnitude of all differences over time are close to zero and are statistically insignificant. Overall, these trends are supportive of the validity of our DiD design. On the other hand, regarding differences in mean values of our outcome variables, we do estimate a statistically significant change in the size of the difference employment from before to during the lockdown period for the probabilities of employment and informal sector employment, but not formal sector employment. This however does not invalidate the validity of our empirical design but instead is indicative of significant and heterogenous treatment effects, which we explore in Sect. 4.

**Fig. 2:**
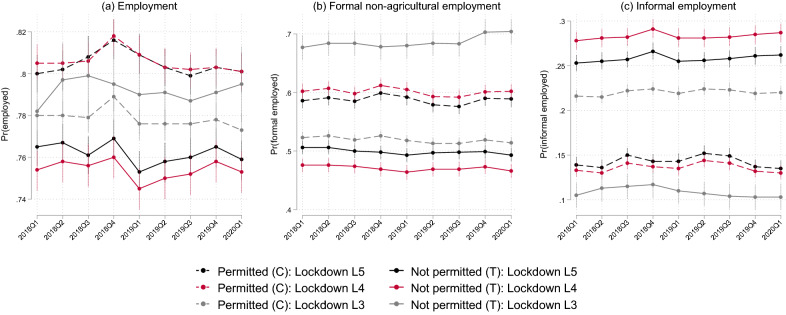
Pre-treatment outcome dynamics, by outcome and lockdown stringency level. *Author’s own calculations. * *Source*: QLFS 2018Q1–2020Q1 (Statistics South Africa 2018a, b, c, d, 2019a, b, c, d, 2020b). *Notes*: This figure presents estimates of trends in mean outcome levels for the treatment (T) (not permitted to work) and control (C) (permitted to work) groups in the pre-treatment period by making use of cross-sectional data from 2018 to 2020. Estimates are presented for each lockdown stringency level which range from level 5 (L5) (most stringent) to L3 (most lenient). Sample restricted to the working-age population (15–64 years). All estimates are weighted using the sampling weights and account for the complex survey design. Spikes represent 95% confidence intervals

As noted above, similar mean levels of covariates between the treatment and control group at baseline is not a requirement in a DiD strategy, but rather what is important is that any observed differences in covariates (but not outcomes) are stable from before the after treatment. Regarding outcomes, it is also important to determine that the two groups are comparable on outcome dynamics in the pre-treatment period. However, such an investigation requires multiple periods in the pre-treatment period. Given that treatment here is based on pre-pandemic industry and that we do not have panel data which precedes 2020Q1, we are unable to accurately conduct such a comparison. However, we are able to make use of weighted *cross-sectional* data from previous waves of the survey to estimate mean outcome levels for the treatment and control groups over time in the pre-treatment period. Although this approach does not make use of the same balanced panel sample used in our modelling approach described above, it seeks to provide some indication that our effect estimates to follow do not simply reflect pre-existing differences between those permitted and not permitted to work. We present these unconditional estimates for nine waves of pre-treatment data in Fig. 2 for each of our three outcome variables and three lockdown stringency levels.

For each outcome and treatment by lockdown level, we find that the estimated trends are indicative that the treatment (not permitted to work) and control (permitted to work) groups are comparable on dynamics in the pre-treatment period. For individuals that would not be permitted to work in the future lockdown level 5, they exhibited a lower overall employment probability (approximately 76% on average) compared to those that would be permitted (80%), as shown in panel (a). Over the period, the levels of these individual probabilities, as well as the between-group difference, changed only marginally in terms of magnitude but not statistical significance. Similar can be said for this group for formal sector employment probability trends, as shown in panel (b). On the other hand, panel (c) shows that those who would not be permitted to work were *more* likely to be employed in the informal sector relative to those that would be permitted. Despite this distinction, the groups follow similar trends: the estimates for each group are relatively constant over time in terms of magnitude and statistical significance. Regarding treatment with respect to lockdown level 4, the differences in overall, formal, and informal sector employment probabilities between those permitted and not permitted to work are similar to their lockdown level 5 counterparts. Finally, regarding treatment with respect to the least stringent lockdown level (3), the inverse holds: individuals that would not be permitted to work in this lockdown level exhibited higher (lower) overall and formal (informal) sector employment probabilities compared to those that would be permitted. Irrespective of this distinction, the pre-treatment outcome dynamics between those permitted and not permitted to work for a given employment type are similar. One exception where these trends do however diverge is in the first and last quarter of 2018 where the estimated probability of overall employment is similar in magnitude for both groups. Despite this, the between-group difference in every wave is statistically insignificant. Overall, these estimates provide some assurance that our treatment and control groups are comparable in outcome dynamics prior to the lockdown period.

### Model specification

In our modelling approach, we estimate effects on employment overall and thereafter explore heterogeneity by employment formality and lockdown stringency. Formally, we estimate the following canonical DiD model specification for individual $i$ in industry $j$ in quarter $t$ using Ordinary Least Squares (OLS):

1 $${y}_{ijt}=\,\alpha +\beta {treatment}_{j}+\delta {post}_{t}+\gamma ({treatment}_{j}\times {post}_{t})+{\mu {\varvec{X}}}_{ijt}+{\varphi }_{i}+{\varepsilon }_{ijt}$$

where ${y}_{ijt}$ is one of three binary employment indicators: employment; formal non-agricultural sector employment; and informal sector employment. Employment is defined as per StatsSA’s conventional definition as working for at least 1 h in the reference week or not working because of temporary absence but have a job to return to. The distinction between formal and informal sector employment we follow here is that followed by Statistics South Africa. Formal non-agricultural sector employment only includes tax-registered workers in all industries excluding agriculture, whereas informal sector employment consists of (i) employees who are not registered for personal income tax and work in establishments that employ fewer than five workers and (ii) employers, the self-employed, and persons helping unpaid in their household business who are not registered for any tax. As implied, this definition is based on two criteria: tax registration status and the size classification of enterprises, whereas the latter criterion only affects the categorization of employees but not other types of workers. Although in the literature tax registration status is often solely used to identify informal sector workers, we are not concerned about the inclusion of this ‘smallness’ criterion for two reasons. First and importantly, the categorisation here follows a two-step process: workers who are not registered for tax are first identified, and only thereafter is data on establishment size used to determine informality status on this subset of workers. Therefore, tax registration status remains the focus or primary criterion while ‘smallness’ is supplemental (Essop and Yu 2008; Fourie and Kerr 2017). Second, as a sensitivity check, we re-estimate all of our models using only the tax registration criterion to define informal sector employment and find near-identical estimates compared to those presented in the next section with respect to the coefficient magnitudes, levels of precision, and levels of statistical significance.^8^

${treatment}_{j}$ is the binary, time-invariant treatment indicator, ${post}_{t}$ indicates whether quarter $t$ is 2020Q2 considering the lockdown commenced at the end of March 2020, and ${\varepsilon }_{ijt}$ is the error term. We control for a vector of time-invariant and time-varying individual-level characteristics, ${\boldsymbol{\rm X}}_{ijt}$, to reduce the residual variance and improve the precision of the estimates, enabling us to rule out a broader range of effect magnitudes. These characteristics include age, sex, racial population group, province of residence, a binary urban residence indicator, and highest education level. We also control employment type which indicates whether an employed individual works for someone else for pay or not, where ‘not’ here includes being an employer, self-employed, or an unpaid household worker, to control for differences in employment characteristics. Following the literature, we avoid controlling for time-varying characteristics which could be considered as outcomes themselves, such as marital status, occupation, and industry; however our estimates are insensitive to their inclusion. We also exploit the unique panel nature of the data to control for ‘industry or occupation job-movers’ (individuals who remain employed over the period but change either occupations or industries at the one-digit level) and ‘formality job movers’ (individuals who remain employed over the period but transition from the formal to informal sector or vice versa). Where ‘formality job movers’ comprise just 2.1% (or 518 unique individuals) of our sample are, 9.6% (or 2360 unique individuals) are ‘industry or occupation job-movers’, while less than 1% are both.^9^ We also exploit the panel to control for individual fixed effects (FE), represented by ${\varphi }_{i}$, which absorb any observable and unobservable time-invariant heterogeneity. When doing so, all time-invariant variables in ${\boldsymbol{\rm X}}_{ijt}$ are automatically omitted from the model, as well as the time-invariant treatment indicator. However, we are still able to estimate $\gamma$—the coefficient of interest—due to prevailing variation in the DiD interaction term induced by within-individual variation in ${post}_{t}$ and between-individual variation in ${treatment}_{j}$ in the pre-treatment period (in other words, prevailing variation in the DiD interaction term). Our approach here is equivalent then to a Two-Way Fixed Effects (TWFE) estimator as referenced in the DiD literature with just two time periods. As such Eq. (1) can be alternatively specified in the following generic function form:

2 $${y}_{ijt}=\alpha +{\varphi }_{i}+{\varphi }_{t}+{\gamma }^{TWFE}{D}_{jt}+{\mu {\varvec{X}}}_{ijt}+{\varepsilon }_{ijt}$$

where ${\varphi }_{t}$ represents time fixed effects and ${D}_{jt}$ is the DiD interaction term. When we examine effect heterogeneity by lockdown stringency, we continue to estimate the above specification but restrict the sample to treatment and control group individuals in a given lockdown level. All standard errors are clustered at the panel (individual) level to allow for correlation in the error for the same individual over time.

## Results

### Employment probabilities

Table 3 presents our specification (1) effect estimates on the probability of employment. By pooling both data periods without controlling for any covariates, the model (1) results suggest that, overall, individuals who were not permitted to work were 4.6 percentage points less likely to be employed—statistically significant at the 1% level. Model (2) shows that this estimate remains similar in both magnitude and statistical significance when we control for data in the lockdown period, and additionally that the average individual was 13.3 percentage points less likely to be employed during this period, regardless of legislated permission-to-work status, which is equivalent to the magnitude of net employment loss observed for the South African labour market during this period. When we control for ${\boldsymbol{\rm X}}_{ijt}$ in model (3), the coefficients of both these variables reduce in magnitude but retain their signs and statistical significance levels. Expectedly, the result of the individual FE model (4) is the omission of the time-invariant treatment variable, but again highlights the significantly lower employment probability of individuals in the lockdown period, regardless of legislated permission-to-work status. Our DiD estimates are presented in models (5)–(7). In our preferred model (7) which controls for ${\boldsymbol{\rm X}}_{ijt}$ and individual FE ${\varphi }_{i}$, we find evidence of a significant and negative lockdown effect. Specifically, our main DiD estimate of interest—$\gamma$—suggests that this core lockdown component decreased the probability of employment for those not permitted to work by just under 3 percentage points relative to those who were permitted to work (in other words, the effect is a reduction of 36.15% relative to the control group), significant at the 1% level. Notably, the magnitude, precision, and statistical significance of the estimated effect are all largely insensitive to the inclusion of observable covariates and individual FE and is similar to the coefficient observed in Column 7 in Table 2.

**Table 3:** Model estimates of lockdown effects on employment probabilities. *Author’s own calculations. Source*: QLFS 2020Q1 and 2020Q2 (Statistics South Africa 2020b, c)

	(1)	(2)	(3)	(4)	(5)	(6)	(7)
	POLS	POLS	POLS	FE	DiD (POLS)	DiD (POLS)	DiD (FE)
Treatment	− 0.046***	− 0.046***	− 0.036***		− 0.030***	− 0.020***	
	(0.008)	(0.008)	(0.007)		(0.008)	(0.008)	
Post		− 0.133***	− 0.123***	− 0.117***	− 0.122***	− 0.111***	− 0.105***
		(0.004)	(0.004)	(0.005)	(0.005)	(0.005)	(0.006)
Treatment × post					− 0.028***	− 0.028***	− 0.029***
					(0.008)	(0.008)	(0.008)
Time-varying controls	N	N	Y	Y	N	Y	Y
Time-invariant controls	N	N	Y	N	N	Y	N
Individual FE	N	N	N	Y	N	N	Y
Constant	0.720***	0.799***	− 0.259***	1.107*	0.792***	− 0.267***	1.079*
	(0.005)	(0.005)	(0.053)	(0.603)	(0.005)	(0.053)	(0.602)
Observations	26,286	26,286	26,069	26,069	26,286	26,069	26,069
R^2^	0.002	0.023	0.193	0.800	0.023	0.193	0.800

This table presents estimates of specification (1) with a binary employment variable serving as the dependent variable. Sample restricted to the working-age population (15–64 years) as of 2020Q1. POLS = pooled ordinary least squares, FE = fixed effects, DiD = Difference-in-Differences. Standard errors presented in parentheses and are clustered at the panel level. All estimates weighted using sampling weights. Time-varying controls include age, highest education level, and employment type. Time-invariant controls include sex, racial population group, province of residence, a binary urban residence indicator, an ‘industry or occupation job-mover’ indicator, and a ‘formality job mover’ indicator as described in Sect. 3.3

***p < 0.01, **p < 0.05, *p < 0.10

### Effect heterogeneity by employment formality

We next investigate whether the estimated lockdown effect above is driven by effects in either the formal or informal sector, or both, in Tables 4 and 5. Beginning with the former, models (1)–(4) reflect findings similar to those for overall employment probabilities as observed in Table 3: overall, individuals who were not permitted to work were less likely to be employed in the formal sector; and during the lockdown period in particular the average individual was less likely to be formally employed, regardless of legislated permission-to-work status, both before and after controlling for ${\boldsymbol{\rm X}}_{ijt}$ and individual FE ${\varphi }_{i}$. Our DiD estimates for formal sector employment in models (5)–(7) are, however, dissimilar from those for overall employment in both magnitude and statistical significance. In our preferred model (7), we do not find any evidence that this core lockdown component had any effect on the probability of formal sector employment for those not permitted to relative to those who were. This estimate is close to zero in magnitude and is not statistically significantly different from zero. This suggests that the significant, negative effect observed for overall employment in Table 3 is not explained by any such effects in the formal sector. Again, the magnitude, precision, and statistical significance of the DiD coefficients are all largely insensitive to the inclusion of observable covariates and individual FE.

**Table 4:** Model estimates of lockdown effects on formal sector employment probabilities. *Author’s own calculations. Source*: QLFS 2020Q1 and 2020Q2 (Statistics South Africa 2020b, c)

	(1)	(2)	(3)	(4)	(5)	(6)	(7)
	POLS	POLS	POLS	FE	DiD (POLS)	DiD (POLS)	DiD (FE)
Treatment	− 0.078***	− 0.079***	− 0.062***		− 0.084***	− 0.068***	
	(0.009)	(0.009)	(0.007)		(0.010)	(0.008)	
Post		− 0.079***	− 0.050***	− 0.055***	− 0.082***	− 0.055***	− 0.053***
		(0.004)	(0.004)	(0.004)	(0.005)	(0.005)	(0.005)
Treatment × post					0.009	0.011	− 0.004
					(0.008)	(0.008)	(0.008)
Time-varying controls	N	N	Y	Y	N	Y	Y
Time-invariant controls	N	N	Y	N	N	Y	N
Individual FE	N	N	N	Y	N	N	Y
Constant	0.535***	0.582***	− 0.637***	1.365**	0.584***	− 0.634***	1.361**
	(0.006)	(0.006)	(0.051)	(0.571)	(0.006)	(0.050)	(0.571)
Observations	26,286	26,286	26,069	26,069	26,286	26,069	26,069
R^2^	0.006	0.012	0.308	0.869	0.012	0.308	0.869

This table presents estimates of specification (1) with a binary formal sector employment variable serving as the dependent variable. Sample restricted to the working-age population (15–64 years) as of 2020Q1. POLS = pooled ordinary least squares, FE = fixed effects, DiD = Difference-in-Differences. Standard errors presented in parentheses and are clustered at the panel level. All estimates weighted using sampling weights. Time-varying controls include age, highest education level, and employment type. Time-invariant controls include sex, racial population group, province of residence, a binary urban residence indicator, an ‘industry or occupation job-mover’ indicator, and a ‘formality job mover’ indicator as described in Sect. 3.3

***p < 0.01, **p < 0.05, *p < 0.10

**Table 5:** Model estimates of lockdown effects on informal sector employment probabilities. *Author’s own calculations. Source*: QLFS 2020Q1 and 2020Q2 (Statistics South Africa 2020b, c)

	(1)	(2)	(3)	(4)	(5)	(6)	(7)
	POLS	POLS	POLS	FE	DiD (POLS)	DiD (POLS)	DiD (FE)
Treatment	0.076***	0.075***	0.067***		0.095***	0.089***	
	(0.006)	(0.006)	(0.006)		(0.008)	(0.007)	
Post		− 0.061***	− 0.081***	− 0.059***	− 0.047***	− 0.066***	− 0.045***
		(0.003)	(0.003)	(0.004)	(0.004)	(0.004)	(0.005)
Treatment × post					− 0.033***	− 0.036***	− 0.035***
					(0.007)	(0.007)	(0.007)
Time-varying controls	N	N	Y	Y	N	Y	Y
Time-invariant controls	N	N	Y	N	N	Y	N
Individual FE	N	N	N	Y	N	N	Y
Constant	0.137***	0.173***	0.249***	0.068	0.165***	0.239***	0.034
	(0.004)	(0.004)	(0.037)	(0.482)	(0.005)	(0.037)	(0.482)
Observations	26,286	26,286	26,069	26,069	26,286	26,069	26,069
R^2^	0.010	0.016	0.188	0.793	0.017	0.189	0.794

This table presents estimates of specification (1) with a binary informal sector employment variable serving as the dependent variable. Sample restricted to the working-age population (15–64 years) as of 2020Q1. POLS = pooled ordinary least squares, FE = fixed effects, DiD = Difference-in-Differences. Standard errors presented in parentheses and are clustered at the panel level. All estimates weighted using sampling weights. Time-varying controls include age, highest education level, and employment type. Time-invariant controls include sex, racial population group, province of residence, a binary urban residence indicator, an ‘industry or occupation job-mover’ indicator, and a ‘formality job mover’ indicator as described in Sect. 3.3

***p < 0.01, **p < 0.05, *p < 0.10

In Table 5, we again examine effect heterogeneity by employment formality but focus on effects on informal sector employment. As opposed to our findings for overall or formal sector employment, models (1)–(3) reflect dissimilar estimates, neither in magnitude nor significance, but in sign. Although we continue to find that, regardless of legislated permission-to-work status, the average individual was less likely to be employed in the informal sector during the lockdown period in particular, we find here that individuals who were not permitted to work were *more* likely to be employed in the informal sector—statistically significant at the 1% level. However, the magnitude and significance of this latter estimate disappears after we control for ${\boldsymbol{\rm X}}_{ijt}$ in model (3). As observed in model (4), after controlling for individual FE ${\varphi }_{i}$ we continue to find that a significantly lower employment probability in the lockdown period, even in the informal sector, regardless of legislated permission-to-work status. Our DiD estimates for informal sector employment in models (5)–(7) are, interestingly, dissimilar from those for overall and formal sector employment, and likely relates to our finding in model (2) that those who were not permitted to work were more likely to be employed in the informal sector. In our preferred model (7), we estimate a significant and negative lockdown effect on informal sector employment of 3.5 percentage points, significant at the 1% level. The magnitude of this estimate is similar to that for overall employment and is not statistically significantly different from it. It should be noted that, again, the magnitude, precision, and statistical significance of this estimate is largely insensitive to the inclusion of observable covariates and individual FE. This, combined with the observed zero effect on formal sector employment in both magnitude and significance, suggests that the significant, negative effect observed for overall employment in Table 3 is driven by a negative employment effect in the informal sector.

### Effect heterogeneity by lockdown stringency

In our analysis above, we find that the significant, negative employment effect of the lockdown is driven not by an effect on formal sector employment but rather on informal sector employment. We next explore effect heterogeneity by varying levels of lockdown stringency for each of our three dependent variables as discussed in Sect. 3. We present the relevant DiD estimates in Table 6, where all models control for both ${\boldsymbol{\rm X}}_{ijt}$ and individual FE ${\varphi }_{i}$. Considering overall employment probabilities in models (1)–(3), we consistently estimate a statistically significant and negative effect, at least at the 5% level. While the magnitude, statistical significance, and precision of the estimated effects for the most stringent lockdown levels (5 and 4) are identical, the estimated effect for the least stringent level (3) is slightly larger in magnitude but exhibits greater uncertainty given the larger standard error and hence lower degree of statistical significance. However, this latter estimate is not statistically significantly different from that of the more stringent levels 5 (*p* = 0.426) or 4 (*p* = 0.467). As such, although we observe negative effects for every level of lockdown stringency, we do not find evidence that these effects vary by lockdown stringency, at least for overall employment probabilities.

**Table 6:** Model estimates of heterogenous lockdown effects on employment probabilities, by formality and lockdown stringency. *Author’s own calculations. Source*: QLFS 2020Q1 and 2020Q2 (Statistics South Africa 2020b, c)

Dependent variable:	(1)	(2)	(3)	(4)	(5)	(6)	(7)	(8)	(9)
	Employment			Formal sector employment			Informal sector employment		
Lockdown level:	5	4	3	5	4	3	5	4	3
Treatment × post	− 0.036***	− 0.038***	− 0.055**	0.008	0.003	− 0.083***	− 0.041***	− 0.053***	0.022
	(0.013)	(0.013)	(0.027)	(0.012)	(0.011)	(0.026)	(0.011)	(0.011)	(0.018)
Time-varying controls	Y	Y	Y	Y	Y	Y	Y	Y	Y
Individual FE	Y	Y	Y	Y	Y	Y	Y	Y	Y
Constant	0.740	0.840	2.230	1.583*	0.763	1.796	− 0.626	0.378	0.956
	(0.909)	(0.993)	(1.357)	(0.863)	(0.934)	(1.261)	(0.759)	(0.771)	(1.078)
Observations	9830	10,116	5978	9830	10,116	5978	9830	10,116	5978
R^2^	0.800	0.801	0.801	0.864	0.870	0.878	0.783	0.796	0.808

This table presents estimates of $\upgamma$ from specification (1) by lockdown level for varying binary dependent variables. Sample restricted to the working-age population (15–64 years) as of 2020Q1. Lockdown levels range from 5 (most stringent) to 3 (most lenient). FE = fixed effects. Standard errors presented in parentheses and are clustered at the panel level. All estimates weighted using sampling weights. ‘Post’ coefficient omitted for brevity. Time-varying controls include age, highest education level, and employment type. Each model controls for individual FEs and as such time-invariant observables are not included

***p < 0.01, **p < 0.05, *p < 0.10

Models (4)–(9) present the relevant heterogeneous effect estimates on formal and informal sector employment probabilities by varying levels of lockdown stringency. Interestingly, our results suggest that informal sector employment is sensitive to higher degrees of lockdown stringency while formal sector employment is sensitive to lower degrees of lockdown stringency. Specifically, we find negative effects of 4.1 percentage points and 5.3 percentage points on the probability of informal sector employment for the most stringent lockdown levels 5 and 4, respectively, both significant at the 1% level. These estimates are not statistically different from one another (*p* = 0.907) and are statistically similar in magnitude to those in models (1) and (2) for overall employment probabilities for the same lockdown levels. However, we do not find evidence of any effect on informal sector employment for the least stringent lockdown level (3)—the coefficient is close to zero in magnitude and is not statistically significant. In contrast, we find no evidence of an effect on the probability of formal sector employment for the most stringent lockdown levels 5 and 4. The magnitudes of both coefficients here are close to zero and are not statistically significant. In contrast, we do however estimate a negative effect of 8.3 percentage points on the probability of formal sector employment for the least stringent lockdown level (3), significant at the 1% level and not statistically different from that in model (3) for the overall probability of employment for the same lockdown level.

### Explaining heterogeneity

Our estimates in this section suggest that while employment probabilities in South Africa were adversely and significantly affected by this core component of the lockdown policy for each of the three lockdown stringency levels assessed here, these effects are heterogeneous by employment formality, where the initial, more stringent lockdown levels negatively affected informal sector employment while the least stringent lockdown level negatively affected formal sector employment. We first hypothesize that these heterogeneous results may be explained by between-sector variation in employment elasticities with respect to, what we refer to as, ‘abrupt’ versus ‘accumulated’ lockdown effects. As described above, the initial ‘hard’ lockdown was implemented quickly and only permitted workers to continue working if they were in occupations deemed essential to economic function or pandemic response, or if they could feasibly work from home. Informal sector workers were less likely to be permitted to work, relative to formal sector workers, and nearly half (46.6%) of all informal sector workers are self-employed in contrast to just 1.9% of formal sector workers.^10^ This suggests that a large proportion of the informal sector had to abruptly cease operations during the most stringent lockdown period. Over time, the easing of lockdown levels eventually permitted almost all sectors to operate thus allowing informal sector activities to resume. By contrast, at the start of the initial stringent lockdown which was at first expected to last for a few weeks, many formal sector workers were shielded from short-term job loss effects through formal employment relationships, a much higher ability to work from home, and access to employment stimulus programmes introduced by the State. As the lockdown persisted, however, formal sector employers’ capacities to retain workers may have waned over time, and hence the job loss effects in this sector may have simply been delayed.

Alternatively, these heterogeneous effects may be explained by a combination of differential targeting and timing of two South African government’s economic support policies during the beginning of the pandemic: the TERS (a wage subsidy) and the COVID-19 SRD grant (an unconditional cash transfer), both described in Sect. 2.2 above. Regarding the former, during the first 2 months (April and May 2020) of the TERS, eligibility was restricted to workers who were registered and contributing to the Unemployment Insurance Fund (UIF) (Köhler et al. 2022). Considering UIF contribution is concentrated in the formal sector in South Africa (as of 2020Q1, 88% of UIF-contributors were formal sector workers),^11^ it can be said that during this period the policy mostly targeted formal sector workers while largely excluding those in the informal sector. Following legal challenges to this eligibility criterion, from the end of May 2020 onwards the policy was expanded to include all workers, whether they were UIF-contributors or non-contributors. This change in eligibility coincides with our employment effect estimates; that is, we find no evidence of any effect on formal sector employment for the most stringent lockdown levels 5 and 4 (in April and May 2020) during which the TERS targeted primarily formal sector workers, but we estimate a negative effect on formal sector employment for the least stringent lockdown level 3 (in June 2020) from which the policy was expanded to include all workers. This then also aligns with our null informal sector employment effect estimates for lockdown level 3 and the negative effects estimated for more stringent levels during which these workers were not eligible. This then suggests that the TERS may have mitigated job loss in the formal sector during its initial period, and once the system expanded to include all workers it then mitigated job loss in the informal sector, possibly at the expense of such mitigation in the formal sector.

A similar story may hold when considering an alternative policy during this period: the COVID-19 SRD grant. Although the policy was announced in April 2020, payments only commenced at the end of May 2020 (Köhler and Bhorat 2021). As such, the grant only largely provided support to the unemployed and informal sector workers (both because the verification process could not distinguish the two groups, as discussed in Sect. 2.2) from June 2020 but not in the two prior months. This aligns with our heterogeneous informal sector employment effect estimates by lockdown stringency; that is, we estimate negative effects for the most stringent lockdown levels 5 and 4 (in April and May 2020) during which the grant did not reach informal sector workers, but we find no evidence of any effects for the least stringent lockdown level 3 (in June 2020). This is indicative that the COVID-19 SRD grant may have mitigated the negative employment effects on informal sector workers once it had rolled out.

Taken together then, a combination of differential targeting and timing of these two core government support policies may explain the heterogeneous employment effects we find. However, given that the QLFS data we use here does not include data on receipt of either policy during the pandemic period, we are unable to conduct an additional empirical analysis and as such cannot make such a conclusion with confidence. An empirical investigation into these mechanisms serves as an important area for future research.

## Robustness tests

**Fig. 3:**
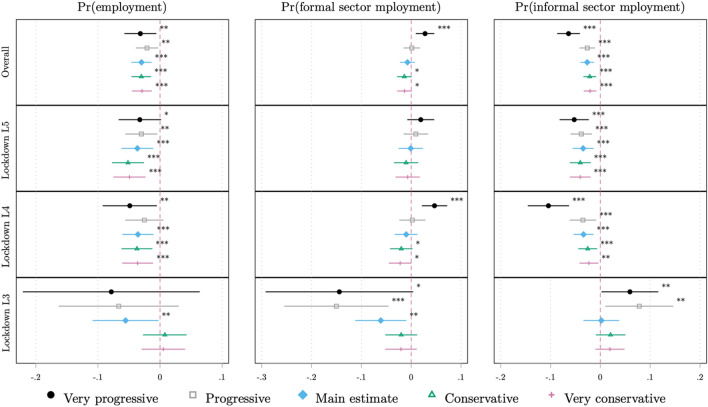
Coefficient plot of model estimates, by outcome, lockdown stringency level, and industry capacity assumption. $\upgamma$ *Author’s own calculations. Source*: QLFS 2020Q1 and 2020Q2 (Statistics South Africa 2020b, c). *Notes*: This figure presents a coefficient plot of estimates of from specification (1) by dependent variable and lockdown level using varying industry capacity assumptions. ‘Very progressive’ = workers coded as being permitted to work if any share of the industry is permitted; ‘progressive’ = workers coded as being permitted to work if at least 25% of the industry is permitted; ‘conservative’ = workers coded as being permitted to work if at least 75% of the industry is permitted; ‘very conservative’ = workers coded as being permitted to work only if 100% of the industry is permitted. Markers represent point estimates and lines represent 95% confidence intervals. Lockdown levels range from 5 (most stringent) to 3 (most lenient). Sample restricted to the working-age population (15–64 years) as of 2020Q1. All model standard errors are clustered at the panel level. Estimated weighted using sampling weights. All models control for a vector of time-varying observable covariates including age, highest education level, and employment type. All models additionally control for individual fixed effects (FEs) and as such time-invariant observables are not included. ***p < 0.01, **p < 0.05, *p < 0.10

In this section, we conduct two robustness tests relating to (1) the assumptions of our empirical strategy and (2) accounting for a possibly confounding covariate. In our main estimation, we assume that individuals who work in ‘limited capacity’ industries were only permitted to work if their industry’s legislated employment capacity was at least 50% and not otherwise. This is an arbitrary threshold and has implications for who is included in our control group. To examine the sensitivity of our results to this decision, we re-estimate specification (1) using four alternative threshold assumptions. The ‘very progressive’ assumption assumes a threshold of 0% (that is, permission-to-work is assumed if an individual’s industry had any legislated capacity above 0%); the ‘progressive’ assumption assumes a threshold of 25%; the ‘conservative’ assumption assumes a threshold of 75%; and under the ‘very conservative’ assumption, permission-to-work is assumed only if 100% of employment within an individual’s industry is permitted. Our main results, which use the ‘50%’ assumption, can be regarded as moderate in this regard. Intuitively, moving from the ‘very progressive’ assumption to the ‘very conservative’ assumption increases the size of our treatment group. This procedure of separately re-estimating specification (1) for each of our dependent variables and levels of lockdown stringency using each of these assumptions results in 60 DiD estimates. We present these estimates, including our main estimates for comparison, in a coefficient plot in Fig. 3.

Considering effects on employment probabilities, for the overall model we consistently estimate a negative and statistically significant effect of a similar magnitude to our main estimate, regardless of assumption. This finding holds when we consider heterogenous effects for lockdown levels 5 and 4, where all estimates are not statistically significantly different from our main estimates and, for the latter lockdown level, the estimates for four out of five alternative assumptions are, at least at the 5% level, statistically significantly different from zero. While for lockdown level 3 only our main estimate is statistically significant, most estimates are negative in sign while two, which are estimated using the ‘very conservative’ and ‘conservative’ assumptions, are close to zero. None of these coefficients are however statistically different from our main estimate. Considering effects on formal sector employment probabilities, for the overall model four of the five coefficients are close to zero and are not significant at least at the 5% level, in line with our main estimate. The ‘very progressive’ estimate represents the exception; however, it is not statistically significantly different from our main estimate, and we believe this assumption—that individuals were permitted to work if their industry had *any* legislated capacity—is very implausible. We find similar results for lockdown level 4, while all estimates for level 5 are not statistically significantly different from zero, in line with our main estimate. For level 3, all five coefficients are negative and, while most (three) are statistically significant, none are statistically significantly different from our main estimate. Finally, considering effects on informal sector employment probabilities, for the overall model we consistently estimate a negative and statistically significant effect by at least the 5% level. This finding holds for both lockdown levels 5 and 4, all in line with our main estimates. For level 3, three of the estimates are not statistically different from zero, in line with our main estimate. The other two, estimated under the ‘very progressive’ and ‘progressive’ assumptions, exhibit a positive coefficient and are statistically significant. However, both are not statistically different from our main estimate. Considering these results, we can conclude that our main estimates largely hold, however some do exhibit a degree of sensitivity to the chosen ‘limited industry’ assumption in a few instances, particularly in the direction of more ‘progressive’ assumptions. However, as previously expressed, we believe these progressive assumptions are not as plausible relative to more moderate assumptions.

Considering South Africa’s five-level risk-adjusted lockdown strategy was in part a function of transmission risk in the workplace (President Ramaphosa 2020), it is possible that our estimated causal effect of the permission-to-work component of the country’s lockdown policy may be confounded by varying task content across occupations, specifically with respect to occupation-specific physical interaction. For instance, workers in occupations which tend to exhibit higher degrees of physical interaction may be less likely to be permitted to work during the lockdown period due to higher transmission risk, implying the potential existence of bias introduced through an omitted variable related to treatment. To account for such an identification threat, we follow Avdiu and Nayyar (2020), Lu (2020), and Bhorat et al. (2020a, b) to construct an occupation-level index of physical interaction (PI), which can be said to measure one aspect of transmission risk, and control for it in a re-estimation of specification (1). Unfortunately, neither the QLFS nor any other existing labour force survey in South Africa includes data on the task content of occupations. As such, to construct our index we merge our data here with occupational work context data from the Occupational Information Network (O*NET), an American survey of detailed occupational information collected by the Bureau of Labour Statistics. We make use of two components from this dataset which are relevant to physical interaction: physical proximity (${P}_{o}$) and face-to-face discussions (${F}_{o}$). Additionally, based on the assumption that workers who use public transport to get to work experience greater physical interaction relative to those using private transport, we additionally merge in work travel data (${T}_{o}$) from StatsSA’s latest Time Use Survey conducted in 2010. Following the Multidimensional Poverty Index literature (Alkire and Foster 2011), these three components are equally-weighted to generate scores for each four-digit level occupation $o$ through the following specification:

3 $$PI_{o}=1/3\,P_{o}+1/3\,F_{o}+1/3\,T_{o}$$

In Table 10 in the “Appendix” we provide information on definitions and the scoring method for each component of the index. Each of the components of the index are scaled to vary within the unit interval such that ${PI}_{o}$ scores vary between 0 and 1 with higher values being indicative of higher levels of physical interaction.^12^ To illustrate the degree of between-industry variation in physical interaction, in Table 9 in the “Appendix” we include estimates of the mean index values by industry. Index values range between 0.118 and 0.934 and exhibit a median of 0.533 and a standard deviation of 0.102, indicative of a non-negligible degree of variation. By industry, physical interaction appears highest in Construction (0.579) and lowest in Private Households (0.457) and Agriculture, hunting, forestry, and fishing (0.467). By formality, physical interaction is higher among formal relative to informal sector workers (0.567 compared to 0.513), and with respect to our treatment groups, physical interaction is higher among those who were not permitted to work relative to those that were (0.564 compared to 0.545, or nearly 20% of a standard deviation), which is in line with our justification for controlling for this measure. Notably, within either the formal or informal sector, physical interaction is higher among those not permitted to work.^13^

After re-estimating specification (1) with the inclusion of this index as a control, our model estimates are presented in Table 7. It should be noted that although this index is time-invariant within occupations during our period here, it is not time-invariant within individuals because some individuals change occupations over time (which we control for using our ‘industry or occupation job-mover’ variable as discussed in Sect. 3.3), and as such we are still able to control for individual FE in our modelling here. The results suggest that our main findings are robust to the inclusion of this covariate in our specification with respect to magnitude, sign, and statistical significance of the estimated effects. Considering effects on employment probabilities, we observe a consistently negative and statistically significant effect overall and for each level of lockdown stringency with a range [− 0.030; − 0.057], none of which are statistically significantly different from our main estimates. For formal sector employment effects, we observe no evidence of any effect overall and for the more stringent lockdown levels 5 and 4, but a statistically significant and negative effect for the least stringent level 3, in line with our main estimates. The results on informal sector employment effects are also in line with our main estimates: a significant and negative effect overall and for the more stringent lockdown levels 5 and 4, but no evidence of any effect for level 3. As a sensitivity test, we alternatively construct the index using Principal Component Analysis (PCA) and find that the estimates are very similar in terms of coefficient magnitude as well as precision (see Table 11 in the “Appendix”). Interestingly, considering panels A and C in Table 7, the coefficient on ${PI}_{o}$ is negative and significant at the 5% level and appears the decrease with lower levels of lockdown stringency, suggesting that informal sector employment is less likely among workers in occupations which exhibit higher levels of physical interaction during periods of high lockdown stringency. We observe no evidence of such a relationship with respect to formal sector employment. In the context of our study here however, this variable is intended solely as an additional control, and as such any further analysis into this relationship is out of this paper’s scope.

**Table 7:** Model estimates, controlling for occupation-level physical interaction. *Author’s own calculations. Source*: QLFS 2020Q1 and 2020Q2 (Statistics South Africa 2020b, c); Time Use Survey 2010 (Statistics South Africa 2014); Occupational Information Network (National Center for O*NET Development 2021)

	Overall	Lockdown level 5	Lockdown level 4	Lockdown level 3
*Panel A: Employment*				
Treatment × post	− 0.030***	− 0.035***	− 0.037***	− 0.057**
	(0.008)	(0.013)	(0.013)	(0.027)
${PI}_{o}$	− 0.156**	− 0.235**	− 0.169*	− 0.004
	(0.069)	(0.111)	(0.096)	(0.155)
Constant	1.946***	1.571*	2.143**	2.639*
	(0.621)	(0.917)	(0.992)	(1.427)
Observations	24,675	9366	9621	5688
*Panel B: Formal sector employment*				
Treatment × post	− 0.006	0.001	− 0.011	− 0.056**
	(0.008)	(0.012)	(0.012)	(0.026)
${PI}_{o}$	0.029	− 0.017	− 0.000	0.210
	(0.067)	(0.116)	(0.094)	(0.138)
Constant	1.510**	2.023**	0.771	1.315
	(0.593)	(0.904)	(0.943)	(1.297)
Observations	24,675	9366	9621	5688
*Panel C: Informal sector employment*				
Treatment × post	− 0.028***	− 0.036***	− 0.035***	− 0.005
	(0.007)	(0.011)	(0.010)	(0.018)
${PI}_{o}$	− 0.155**	− 0.195*	− 0.141*	− 0.201
	(0.062)	(0.107)	(0.081)	(0.133)
Constant	0.313	− 0.703	1.422*	0.960
	(0.485)	(0.718)	(0.775)	(1.133)
Observations	24,675	9366	9621	5688

This table presents estimates of Eq. (1), overall and by lockdown level, for varying binary dependent variables while additionally controlling for occupation-level workplace physical interaction. Sample restricted to the working-age population (15–64 years) as of 2020Q1. Lockdown levels range from 5 (most stringent) to 3 (most lenient). All models control for a vector of time-varying observable covariates including age, highest education level, and employment type, as well as individual fixed effects (FEs). PI index = Physical Interaction index generated by merging occupation-level Occupational Information Network (O*NET) data and Time Use Survey 2010 with the QLFS data. Standard errors presented in parentheses and are clustered at the panel level. Estimates weighted using sampling weights. ‘Post’ coefficient omitted for brevity

***p < 0.01, **p < 0.05, *p < 0.10

## Conclusion

Like many governments around the world, the South African government implemented a national lockdown in response to the COVID-19 pandemic to prepare necessary health infrastructure as well as delay and minimise the spread of the virus. This initial lockdown was stringent by international standards and official labour force data revealed significant job losses equivalent to the total number of net jobs created over the previous decade. However, as observed in other economies, the distribution of job loss during this initial period was not uniform but instead heavily skewed by employment formality towards those working in the informal sector.

Importantly, lockdown policy is not time-invariant, and varying levels of lockdown stringency over time shape the nature of job losses across different labour market sub-groups. Although studies providing evidence on the causal labour market effects of the pandemic and lockdown policies exist in both developed and developing countries, there is a lack of causal evidence on how variation in lockdown stringency affects labour market outcomes, particularly in developing countries. Such variation may have heterogenous effects on aggregate and by employment formality. From a policy perspective, evidence of such heterogeneity can inform decisions around the optimal targeting of government support as the pandemic progresses and lockdown policies are reconsidered.

In this paper, we estimate the causal effect of lockdown stringency on employment probabilities and examine effect heterogeneity by employment formality by making use of representative, individual-level, panel labour force data and adopting a quasi-experimental econometric design which exploits temporal variation in employment probabilities of adults who were and were not permitted to work. We do so in the context of South Africa—a useful case study given that as an upper-middle-income country with a relatively small informal sector employment share, our findings may be broadly useful to both developing countries (given South Africa’s level of economic development) as well as to more developed countries (given South Africa’s low informal sector employment share). We find that South Africa’s lockdown policy significantly reduced the overall probability of employment at every level of lockdown stringency, but these effects were driven by negative employment effects in the informal sector. Notably, we find notably effect heterogeneity by employment formality, where more stringent lockdown levels had large negative effects on informal sector employment, but not formal sector employment. By contrast, the least stringent lockdown level negatively affected formal sector employment but not informal sector employment. These results hold when subject to robustness tests that control for varying task content across occupations as well as varying treatment group assumptions.

We put forward two hypotheses for the heterogeneous relationship we find between lockdown stringency and formality of employment. First, these findings may be related to between-sector variation in employment elasticities with respect to ‘abrupt’ versus ‘accumulated’ lockdown effects. Second, it is plausible that these findings may be explained by a combination of differential targeting and timing of two of the government’s core economic support policies during the beginning of the pandemic: a wage subsidy which temporarily targeted primarily formal sector workers and a new unconditional cash transfer which provided support to informal sector workers, but which experienced a delayed rollout. The differential timing and targeting of these policies coupled with our findings suggest that they may have mitigated the negative employment effects of the country’s lockdown policy. However, a detailed analysis of these mechanisms lies beyond the scope of this paper and as such we are unable to make conclusive statements in this regard, but such an empirical analysis serves as an important area for future research.

In summary, our analysis provides empirical evidence on the differential effects of lockdown policies by level of stringency and employment formality in a large developing country economy. As governments continue to consider lockdown regulations as a policy response, whether to the COVID-19 pandemic or a future crisis, policymakers ought to be mindful of the existence of such heterogeneous effects by lockdown stringency and employment formality in their efforts to target government support appropriately.

## Data Availability

The datasets produced by Statistics South Africa analysed in this study are available in the DataFirst repository, available here: https://www.datafirst.uct.ac.za/dataportal/. However, the Quarterly Labour Force Survey 2020 Quarter 2 dataset in the public domain does not include interview month data which was provided to us by Statistics South Africa, but is available from the corresponding author on reasonable request and with permission from Statistics South Africa. The datasets produced by the National Center for O*NET Development analysed in this study are available in the O*NET repository, available here: https://www.onetonline.org/. The analysis was conducted using Stata.

## Appendix

See Tables 8, 9, 10 and 11.

**Table 8:** Sample sizes and weighted population estimates, by year and quarter. *Author’s own arrangement. **Source*: QLFS 2019Q1, 2019Q2, 2020Q1, and 2020Q2 (Statistics South Africa 2019a, b, 2020b, c)

	2019Q1		2019Q2		2020Q1		2020Q2	
	N	Weighted estimate	N	Weighted estimate	N	Weighted estimate	N	Weighted estimate
Total	67,480	57,071,059	67,626	57,251,253	66,657	57,792,395	47,103	57,973,917
		(548,901)		(551,886)		(575,263)		(803,347)
Working-age population	42,024	38,282,909	42,210	38,432,975	41,827	38,873,945	29,495	39,021,017
		(379,362)		(380,014)		(393,488)		(547,142)
Employed	17,490	16,291,436	17,414	16,312,706	17,044	16,382,555	10,001	14,148,215
		(185,895)		(191,220)		(196,425)		(246,679)
Unemployed (broad definition)	10,959	9,994,457	11,244	10,226,485	11,577	10,796,924	7624	10,259,336
		(161,116)		(160,964)		(169,914)		(221,937)
Economically inactive (broad definition)	13,575	11,997,016	13,552	11,893,784	13,206	11,694,466	11,870	14,613,465
		(178,093)		(171,581)		(168,282)		(263,776)

This table presents, for each quarter in the data, sample sizes (N) and weighted population estimates of given groups of individuals by year and quarter (Q). Relevant estimates weighted using sampling weights. Labour market groups restricted to the working age (15–64 years). Standard errors presented in parentheses and account for the complex survey design

**Table 9:** Industry-level variation in legislated permission to work, by lockdown level. *Author’s own arrangement. **Source*: COVID-19 Risk Adjusted Strategy (Department of Health 2020); QLFS 2020Q1 and 2020Q2 (Statistics South Africa 2020b, c); Time Use Survey 2010 (Statistics South Africa 2014); Occupational Information Network (National Center for O*NET Development 2021)

SIC code	Industry description	Level 5	Level 4	Level 3	Mean PI index
*10*	*Private households*				*0.457*
10	Private households with employed persons	0	0	1	0.457
*11*	*Other*				*0.477*
20	Exterritorial organisations	1	1	1	0.453
30	Representatives of foreign governments	1	1	1	0.500
*1*	*Agriculture, forestry, and fishing*				*0.510*
111	Growing of crops; market gardening; horticulture	1	1	1	0.465
112	Farming of animals	1	1	1	0.470
113	Growing of crops combined with farming of animals (mixed farming)	1	1	1	0.451
114	Agricultural and animal husbandry services, except veterinary activities	1	1	1	0.449
115	Hunting, trapping and game propagation, including related services	1	1	1	0.382
116	Production of organic fertilizer	1	1	1	0.514
121	Forestry and related services	0	1	1	0.467
122	Logging and related services	0	1	1	0.505
131	Ocean and coastal fishing	1	1	1	0.591
132	Fish hatcheries and fish farms	1	1	1	0.806
*2*	*Mining and Quarrying*				*0.581*
210	Mining of coal and lignite	1	1	1	0.564
230	Mining of gold and uranium ore	0.25	0.5	1	0.577
240	Mining of metal ores, except gold and uranium	0.25	1	1	0.645
241	Mining of iron ore	0.25	1	1	0.560
242	Mining of non-ferrous metal ores, except gold and uranium	0.25	0.5	1	0.569
251	Stone quarrying, clay and sandpits	0.25	1	1	0.568
252	Mining of diamonds	0.25	1	1	0.615
253	Mining and quarrying not elsewhere classified	0.25	0.5	1	0.547
*3*	*Manufacturing*				*0.557*
301	Production, processing and preservation of meat, fish, fruit, vegetables, oils and fats	1	1	1	0.616
302	Manufacture of dairy products	1	1	1	0.575
303	Manufacture of grain mill products, starches and starch products and prepared animal feeds	1	1	1	0.610
304	Manufacture of other food products	1	1	1	0.569
305	Manufacture of beverages	0.8	0.8	1	0.530
306	Manufacture of tobacco products	1	1	1	0.554
311	Spinning, weaving and finishing of textiles	0.25	0.5	1	0.591
312	Manufacture of other textiles	0.25	0.5	1	0.535
313	Manufacture of knitted and crocheted fabrics and articles	0.25	0.5	1	0.583
314	Manufacture of wearing apparel, except fur apparel	0.25	0.5	1	0.519
316	Tanning and dressing of leather; manufacture of luggage, handbag, saddlery and harness	0.25	0.5	1	0.504
317	Manufacture of footwear	0.25	0.5	1	0.573
321	Sawmilling and planing of wood	0	0.5	1	0.526
322	Manufacture of products of wood, cork, straw and plaiting materials	0	0.5	1	0.584
323	Manufacture of paper and paper products	1	1	1	0.550
324	Publishing	0	0.5	1	0.485
325	Printing and service activities related to printing	0	0.5	1	0.482
332	Petroleum refineries and synthesisers	1	1	1	0.585
333	Processing of nuclear fuel	1	1	1	0.488
334	Manufacture of basic chemicals	0	0.2	1	0.588
335	Manufacture of other chemical products	0	0.2	1	0.577
337	Manufacture of rubber products	0	0.5	1	0.524
338	Manufacture of plastic products	1	1	1	0.587
341	Manufacture of glass and glass products	1	1	1	0.577
342	Manufacture of non-metallic mineral products not elsewhere classified	0	0.2	1	0.547
351	Manufacture of basic iron and steel	0	0.5	1	0.563
352	Manufacture of basic precious and non-ferrous metals	0	0.5	1	0.571
354	Manufacture of structural metal products, tanks, reservoirs and steam generators	0	0.5	1	0.537
355	Manufacture of other fabricated metal products; metalwork service activities	0	0.5	1	0.532
356	Manufacture of general purpose machinery	0	0.5	1	0.566
357	Manufacture of special purpose machinery	0	0.5	1	0.538
358	Manufacture of household appliances not elsewhere classified	0	0.2	1	0.599
359	Manufacture of office, accounting and computing machinery	0	0.2	1	0.581
361	Manufacture of electric motors, generators and transformers	0	0.2	1	0.550
362	Manufacture of electricity distribution and control apparatus	1	1	1	0.600
363	Manufacture of insulated wire and cable	0	0.2	1	0.584
364	Manufacture of accumulators, primary cells and primary batteries	0	0.2	1	0.557
365	Manufacture of electric lamps and lighting equipment	0	0.2	1	0.650
366	Manufacture of other electrical equipment not elsewhere classified	0	0.2	1	0.551
371	Manufacture of electronic valves and tubes and other electronic components	0	0.2	1	0.604
372	Manufacture of television and radio transmitters and apparatus for line telephony and line telegraphy	0	0.2	1	0.460
374	Manufacture of medical appliances and instruments and appliances for measuring, checking, testing, navigating and for other purposes, except optical instruments	1	1	1	0.557
375	Manufacture of optical instruments and photographic equipment	0	0.2	1	0.521
381	Manufacture of motor vehicles	0	0.5	1	0.554
382	Manufacture of bodies (coachwork) for motor vehicles; manufacture of trailers and semi-trailers	0	0.5	1	0.572
383	Manufacture of parts and accessories for motor vehicles and their engines	0	0.5	1	0.563
384	Building and repairing of ships and boats	0	0.2	1	0.620
385	Manufacture of railway and tramway locomotives and rolling stock	0	0.2	1	0.549
386	Manufacture of aircraft and space-craft	0	0.2	1	0.545
387	Manufacture of transport equipment not elsewhere classified	0	0.2	1	0.519
391	Manufacture of furniture	0	0.2	1	0.548
392	Manufacturing not elsewhere classified	0	0.2	1	0.573
395	Recycling not elsewhere classified	0	0.2	1	0.511
*4*	*Electricity; gas and water supply*				*0.559*
411	Production, collection and distribution of electricity	1	1	1	0.582
412	Manufacture of gas; distribution of gaseous fuels through mains	1	1	1	0.512
420	Collection, purification, and distribution of water	1	1	1	0.582
*5*	*Construction*				*0.545*
501	Site preparation	0.1	0.25	0.5	0.459
502	Building of complete constructions or parts thereof; civil engineering	0.1	0.25	0.5	0.573
503	Building installation	0.1	0.25	0.5	0.600
504	Building completion	0.1	0.25	0.5	0.589
505	Renting of construction of demolition equipment with operators	0.1	0.25	0.5	0.505
*6*	*Wholesale and retail trade*				*0.580*
611	Wholesale trade on a fee or contract basis	0.1	0.25	0.5	0.564
612	Wholesale trade in agricultural raw materials, livestock, food, beverages and tobacco	1	1	1	0.608
613	Wholesale trade in household goods	1	1	1	0.658
614	Wholesale trade in non-agricultural intermediate products, waste and scrap	1	1	1	0.553
615	Wholesale trade in machinery, equipment and supplies	0.1	0.25	1	0.490
619	Other wholesale trade	0.1	0.25	0.5	0.558
621	Non-specialised retail trade in stores	0.1	0.25	0.5	0.642
622	Retail trade in food, beverages and tobacco in specialised stores	0.75	0.8	0.9	0.619
623	Other retail trade in new goods in specialised stores	0.1	0.25	0.5	0.602
624	Retail trade in second-hand goods in stores	0.1	0.25	0.5	0.650
625	Retail trade not in stores	1	1	1	0.459
626	Repair of personal and household goods	0.1	0.25	1	0.560
631	Sale of motor vehicles	0	0	0.5	0.574
632	Maintenance and repair of motor vehicles	0.1	0.25	0.5	0.496
633	Sale of motor vehicle parts and accessories	0.1	0.25	0.5	0.578
634	Sale, maintenance and repair of motor cycles and related parts and accessories	0.1	0.25	0.5	0.609
635	Retail sale of automotive fuel	1	1	1	0.646
641	Hotels, camping sites and other provision of short-stay accommodation	0.1	0.1	0.1	0.553
642	Restaurants, bars and canteens	0	0.25	0.25	0.642
643	Shebeen	0	0	0.1	0.547
*7*	*Transport, storage and communication*				*0.562*
711	Railway transport	0.6	0.6	0.6	0.572
712	Other land transport	0.1	0.25	0.5	0.580
721	Sea and coastal water transport	0.1	0.25	0.5	0.513
730	Air transport	0.1	0.25	0.5	0.582
741	Supporting and auxiliary transport	0.1	0.25	0.5	0.588
751	Postal and related courier activities	0.1	0.1	0.25	0.584
752	Telecommunication	1	1	1	0.516
*8*	*Finance*				*0.501*
811	Monetary intermediation	1	1	1	0.526
818	Cash loans	1	1	1	0.491
819	Other financial intermediation not elsewhere classified	1	1	1	0.468
821	Insurance and pension funding, except compulsory social security	1	1	1	0.475
831	Activities auxiliary to financial intermediation, except insurance and pension funding	1	1	1	0.449
841	Real estate activities with own or leased property	0	0	0.5	0.408
842	Real estate activities on a fee or contract basis	0	0	0.5	0.453
851	Renting of transport equipment	0.4	0.6	1	0.555
852	Renting of other machinery and equipment	0.4	0.6	1	0.495
853	Renting of personal and household goods not elsewhere classified	0.4	0.6	1	0.517
862	Software consultancy and supply	1	1	1	0.471
863	Data processing	1	1	1	0.521
864	Data base activities	1	1	1	0.513
865	Maintenance and repair of office, accounting and computing machinery	0	0	0.25	0.504
869	Other computer related activities	1	1	1	0.542
871	Research and experimental development on natural sciences and engineering	1	1	1	0.519
881	Legal, accounting, bookkeeping and auditing activities; tax consultancy; market research and public-opinion research; business and management consultancy	1	1	1	0.457
882	Architectural, engineering and other technical activities	1	1	1	0.534
883	Advertising	1	1	1	0.543
889	Business activities not elsewhere classified	1	1	1	0.582
*9*	*Community, social, and personal services*				*0.544*
911	Central government activities	1	1	1	0.519
913	Local authority activities	1	1	1	0.528
914	Provincial administrations	1	1	1	0.500
915	SA Defence force	1	1	1	0.533
916	SA Police service	1	1	1	0.580
917	Correctional service	1	1	1	0.585
920	Education	0.1	0.1	0.25	0.605
931	Human health activities	1	1	1	0.620
932	Veterinary activities	1	1	1	0.575
933	Social work activities	0.5	1	1	0.597
940	Sewage and refuse disposal, sanitation and similar activities	1	1	1	0.487
951	Activities of business, employers and professional organisations	0.1	0.25	0.5	0.473
952	Activities of trade unions	0.1	0.25	0.5	0.516
959	Activities of other membership organisations	0	0	0	0.445
961	Motion picture, radio, television and other entertainment activities	0	0	0	0.519
962	News agency activities	1	1	1	0.495
963	Library, archives, museums and other cultural activities	0	0	0	0.545
964	Sporting and other recreational activities	0	0	0	0.585
990	Other service activities	0.1	0.25	0.5	0.621

This table presents a list of industries using Standard industrial Classification (SIC) codes that were and were not permitted to work by legislation for each of South Africa’s lockdown levels during the period of study (levels 5, 4, and 3). In columns (3)–(5), 1 = permitted to work; 0 = not permitted to work. If a given industry was permitted to work but at a limited employment capacity, (0; 1) = the capacity in proportional terms. Column (6) presents the mean level of physical interaction for each industry according to the physical interaction index as described in Sect. 5. The values in italics denotes major industry groups or subheadings

**Table 10:** Physical interaction index component definitions. Author’s own arrangement

Component	Definition	Scoring method	Source
Physical proximity (${P}_{o}$)	1. I don't work near other people (beyond 100 ft.)	O*NET spreads 100 points across five levels per occupation. Our approach multiplies points by their category level and sums to get a score. We sum points in categories 3–5 only to reach a score out of 500 (the maximum feasible score). We rescale this to vary [0; 1]	O*NET
	2. I work with others but not closely (e.g., private office)		
	3. Slightly close (e.g., shared office)		
	4. Moderately close (at arm's length)		
	5. Very close (near touching)		
Face-to-face discussions (${F}_{o}$)	1. Never	O*NET spreads 100 points across five levels per occupation. Our approach multiplies points by their category level and sums to get a score. We sum points in categories 4–5 only to reach a score out of 500 (the maximum feasible score). We rescale this to vary [0; 1]	
	2. Once a year or more but not every month		
	3. Once a month or more but not every week		
	4. Once a week or more but not every day		
	5. Every day		
Public transport (${T}_{o}$)	Ever used any type of public transport to travel to work on a given day	Share per occupation. Varies [0, 1]	StatsSA Time Use Survey 2010
	Private transport is defined as walking, cycling, or private vehicle		
	Public transport is defined as bus, taxi, train, or other transport		

This table presents a description of the three components of our occupation-level Physical Interaction index ${\mathrm{PI}}_{\mathrm{o}}$ created using data from the Occupational Information Network (O*NET) and StatsSA’s latest Time Use Survey conducted in 2010

**Table 11:** Model estimates, controlling for occupation-level physical interaction constructed using principal component analysis. *Author’s own calculations. **Source*: QLFS 2020Q1 and 2020Q2 (Statistics South Africa 2020b, c); Time Use Survey 2010 (Statistics South Africa 2014); Occupational Information Network (National Center for O*NET Development 2021)

	Overall	Lockdown level		
		5	4	3
*Panel A: Employment*	(1)	(2)	(3)	(4)
Treatment × post	− 0.030***	− 0.035***	− 0.037***	− 0.057**
	(0.008)	(0.013)	(0.013)	(0.027)
${PI}_{o}^{PCA}$	− 0.007	− 0.008	− 0.018*	0.011
	(0.007)	(0.011)	(0.010)	(0.015)
Constant	1.868***	1.440	2.039**	2.644*
	(0.621)	(0.917)	(0.993)	(1.426)
Observations	24,678	9368	9621	5689
*Panel B: Formal employment*				
Treatment × post	− 0.006	0.002	− 0.011	− 0.057**
	(0.008)	(0.012)	(0.012)	(0.026)
${PI}_{o}^{PCA}$	0.020***	0.026**	0.007	0.035***
	(0.006)	(0.011)	(0.009)	(0.013)
Constant	1.547***	2.039**	0.784	1.413
	(0.592)	(0.904)	(0.944)	(1.297)
Observations	24,678	9368	9621	5689
*Panel A: Informal employment*				
Treatment × post	− 0.028***	− 0.035***	− 0.034***	− 0.004
	(0.007)	(0.011)	(0.010)	(0.018)
${PI}_{o}^{PCA}$	− 0.022***	− 0.029***	− 0.021***	− 0.021
	(0.006)	(0.009)	(0.008)	(0.013)
Constant	0.217	− 0.833	1.325*	0.873
	(0.487)	(0.728)	(0.776)	(1.128)
Observations	24,678	9368	9621	5689

This table presents estimates of Eq. (1), overall and by lockdown level, for varying binary dependent variables while additionally controlling for occupation-level workplace physical interaction, similar to Table 7 but instead here the index is constructed using Principal Component Analysis. Sample restricted to the working-age population (15–64 years) as of 2020Q1. Lockdown levels range from 5 (most stringent) to 3 (most lenient). All models control for a vector of time-varying observable covariates including age, highest education level, and employment type, as well as individual fixed effects (FEs). PI index = Physical Interaction index generated by merging occupation-level Occupational Information Network (O*NET) data and Time Use Survey 2010 with the QLFS data. Standard errors presented in parentheses and are clustered at the panel level. Estimates weighted using sampling weights. ‘Post’ coefficient omitted for brevity

****p* < 0.01, ***p* < 0.05, **p* < 0.10

## Footnotes

1 See Béland et al. (2020), Gupta et al. (2020), and Baek et al. (2021) for the United States; Aum et al. (2021) for South Korea; Born et al. (2021) for Sweden; and Betcherman et al. (2020) for Greece.

2 See Schotte et al. (2021) for Ghana.

3 South Africa’s informal sector accounts for less than 25% of total employment, compared to an average of 70% in developing and emerging countries and 89.2% in Sub-Saharan Africa (Bonnet et al. 2019).

4 The stringency index is calculated as the mean value of the nine metrics, each of which vary between 0 and 100. See Hale et al. (2021) for more information on the methodology.

5 However, the government gazettes made provision for deviation from the phased-return approach based on a school’s ability to comply with COVID-19 guidelines and approval by the provincial Head of Department. Mohohlwane et al. (2021) show positive attendance rates for children in grades that were not yet officially open in a given period, and suggest that this is possibly because some schools, especially private schools, did apply for permission to have other grades return, and some schools made attempts to provide remote schooling through online platforms.

6 Additionally, amongst those who DiD have contact numbers, some contact numbers were found to be invalid or were not answered during data collection, and some households indicated that they were no longer residing at the dwelling units they had occupied during 2020Q1. All of these were regarded as non-contact and were adjusted for during the weighting processes.

7 It should be noted that a new theoretical and empirical literature on the econometrics of DiD has developed over the last few years, with most studies highlighting how in practice typical applications do not meet all requirements of the canonical DiD setup due to the presence of more than two time periods and heterogenous or ‘staggered’ treatment timing [see Roth et al. (2022) for a recent review]. In our study here however, we only have two periods, and every treated observation is treated at the same period (2020Q2). As such, we proceed with the canonical setup.

8 We do not present these results in this paper; however, we are happy to provide them upon request.

9 On average and relative to those who remain in the same sector, ‘formality job movers’ are older (39.5 years versus 36.3 years), more likely to be men (61% vs. 45%), live in urban areas (72% vs. 64%), and have at least completed a secondary education level or equivalent (49% vs. 41%). On average and relative to those who do not switch industry or occupation, ‘industry or occupation job-movers’ are older (41 vs. 36 years), more likely to be men (56% vs. 44%), live in urban areas (78% vs. 63%), and have at least completed a secondary education level or equivalent (62% vs. 39%). All these between-sample differences are statistically significant by at least the 5% level.

10 Own calculations using microdata from the Quarterly Labour Force Survey for the first quarter of 2020 (Statistics South Africa 2020b).

11 Own calculations using weighted microdata from StatsSA’s QLFS for 2020Q1.

12 We adjust the generated score for one occupation to ensure the American O*NET data is relevant for the South African context. The initial scoring resulted in domestic workers exhibiting a low ${PI}_{o}$ value driven by a low ${P}_{o}$ score. However, in South Africa domestic workers often perform a dual role of cleaning and child-minding (du Plessis 2018), leading us to believe this low physical proximity score was inappropriate for the South African context. We adjusted the ${P}_{o}$ score for this occupation by replacing it with the mean of the ${P}_{o}$ score for domestic workers and the ${P}_{o}$ score for child-care workers.

13 Within the formal sector, the physical interaction index is 0.583 for workers who were not permitted to work in a given lockdown level compared to 0.558 for those who were. Within the informal sector, the physical interaction index is 0.522 for workers who were not permitted to work in a given lockdown level compared to 0.503 for those who were.
